# Multiple comparisons analysis of serological data from an area of low *Plasmodium falciparum* transmission

**DOI:** 10.1186/s12936-015-0955-1

**Published:** 2015-11-04

**Authors:** Eric Rogier, Ryan Wiegand, Delynn Moss, Jeff Priest, Evelina Angov, Sheetij Dutta, Ito Journel, Samuel E. Jean, Kimberly Mace, Michelle Chang, Jean Frantz Lemoine, Venkatachalam Udhayakumar, John W. Barnwell

**Affiliations:** Malaria Branch, Division of Parasitic Diseases and Malaria, Centers for Disease Control and Prevention, Center for Global Health, Atlanta, GA USA; Malaria Vaccine Branch, Walter Reed Army Institute of Research, Silver Spring, MD USA; Laboratoire National de Santé Publique (LNSP)/Ministère de la Santé Publique et de la Population (MSPP), Port-au-Prince, Haiti; Population Services International/Organisation Haïtienne de Marketing Social pour la Santé, Port-au-Prince, Haiti; Programme National de Contrôle de la Malaria/MSPP, Port-au-Prince, Haiti

**Keywords:** Serology, *Plasmodium falciparum*, Multiplex, ELISA, Distribution

## Abstract

**Background:**

As a nation reduces the burden of falciparum malaria, identifying areas of transmission becomes increasingly difficult. Over the past decade, the field of utilizing malaria serological assays to measure exposure has grown rapidly, and a variety of serological methods for data acquisition and analysis of human IgG against falciparum antigens are available. Here, different immunoassays and statistical methods are utilized to analyse samples from a low transmission setting and directly compare the estimates generated.

**Methods:**

A subset of samples (n = 580) from a 2012 Haitian nationwide malaria survey was employed as sample population of low falciparum endemicity. In addition to the Haitian samples, samples from 247 US residents were used as a reference population of ‘true seronegatives’. Data acquisition was performed through standard ELISA and bead-based multiplex assays assaying for IgG antibodies to the *Plasmodium falciparum* antigens MSP-1p19, MSP-1p42(D), MSP-1p42(F), and AMA-1. Appropriate parametric distributions and seropositivity cutoff values were determined by statistical measures.

**Results:**

Data from both assays showed a strong positive skew, and the lognormal distribution was found to be an appropriate statistical fit to the Haitian and American populations. The American samples served as a good serological true negative population for the multiplex assay, but not for ELISA-based data. Mixture model approaches to determine seronegative and seropositive populations from the Haitian data showed a high degree of distribution overlap—likely due to the historical low falciparum transmission in this nation. Different fittings to the reversible catalytic model resulted depending upon the immunoassay utilized and seropositivity cutoff method employed. Data were also analysed through fitting to penalized B-splines, presenting another possible analytical tool for the analysis of malaria serological data.

**Conclusions:**

Standardization of serological techniques and analyses may prove difficult as some tools can prove to be more useful depending on the area and parasite in question, making clear interpretation a vital pursuit. The presented analysis in the low-endemic nation of Haiti found malaria-naive US residents to be an appropriate seronegative reference population for the multiplex assay, and this assay providing consistent estimates between MSP-1 and AMA-1 antigens of percent seropositives for this low-endemic population.

**Electronic supplementary material:**

The online version of this article (doi:10.1186/s12936-015-0955-1) contains supplementary material, which is available to authorized users.

## Background

Malaria caused by the apicomplexan parasite *Plasmodium falciparum* remains a significant global health concern with approximately 200 million cases and 600,000 deaths annually [1]. With the enormous burden *P. falciparum* has placed on humans and their ancestors, it is rightfully stated that “malaria is the strongest known selective pressure in the recent history of the human genome” [2]. Besides the manipulations to the structure of haemoglobin as a strategy to prevent malaria death, the human genome has also adapted to recognize numerous *P. falciparum* antigens as targets for a humoral response. Many of the most immunogenic antigens include membrane bound proteins that are found on the surface of invasive merozoites, which are released from infected erythrocytes following schizont-induced rupture of the host cell.

One of the important factors that determines an individual’s carriage of memory B cells educated against *P. falciparum* antigens (and the serum IgG specific for these antigens) is age. If sustained transmission, no matter how low, is present in a geographical area, persons in that area have a greater cumulative risk of lifetime exposure as they age. In regions of moderate or high transmission for *P. falciparum*, children progressing through their first decade of life show rapid increases in the acquisition of antibodies against the parasite [3–5]. The rate of serological development has been modelled by employing a reversible catalytic conversion model [6], which provides estimates for a population’s rates of antibody acquisition (seroconversion) as well as loss (seroreversion). The seroconversion rate for any particular antigen is due, in part, to the half-life of the memory B cell specific for the antigen, and it has been hypothesized that as age and exposure increase, so does the half-life and quantity of B cells specific for an antigen [7, 8]. A peculiar finding by many groups has shown that even in historically high transmission zones, older age groups do not necessarily carry appreciable levels of long-lived IgG against *P. falciparum* [5, 9], even though multiple lifetime infections would have been nearly certain. Although the true explanation for this observation is likely multifaceted, one possibility involves the loss of antibodies over time through seroreversion [8].

As malaria incidence within an area decreases, the ability to detect active infections becomes increasingly difficult. The reduction of *P. falciparum* biomass within an area has been shown to relegate infections much more heavily towards sub-patent, sub-microscopic asymptomatic presentations [10–12]. For nations initiating pre-elimination programmes, this greatly reduces the efficacy of transmission zone discovery through passive case detection [13]. Sensitive, nucleic acid-based technologies exist for the detection of low-parasitaemic infections, but are expensive, impractical for large sample sizes, and have been shown to vary widely in their lower limits-of-detection based on protocols and operators [9]. Furthermore, the window of time an individual could test positive is brief and based solely on a considerable amount of circulating parasites. More recent efforts have attempted to use serological markers as a proxy to estimate *P. falciparum* transmission intensity in areas with low parasite prevalence [6, 14, 15].

Many strategies have been utilized for the analysis and interpretation of malaria serological data. Unlike active infection detection which primarily focuses only on the presence or absence of infection, serological datasets can provide a wealth of information, especially when combined with other covariates such as age. Recently, quantitative immunoassays, such as ELISA and bead-based flow assays, have been the tests of choice, allowing relatively short sample preparation time and robust data generation. In addition, multiple methods have been employed with efforts to maintain the continuous nature of the data as well as dichotomize the sample population into classes of ‘seropositive’ or ‘seronegative’. When dichotomizing variables, further various approaches have been used in order to determine an assay value at which the data can be partitioned—generally referred to as the cutoff value. Here is presented analyses of serological data from a region of low *P. falciparum* endemicity by comparison of immunoassays, variable classifications, and cutoff value determinations. Data is also presented from a large sample of persons never exposed to malaria to show IgG distributions in a malaria-naive population.

## Methods

### Ethical considerations

The survey protocol was approved by CDC’s internal review board and the Haiti ethical review committee in 2012 at the Ministry of Health. All persons participating in the survey were assigned a six-digit identification number that could not be traced back to the individual.

### Survey design and sample collection

Samples from persons assumed to have never been infected with malaria were gathered from blood donated to a community blood bank in Memphis, TN, USA. All blood units were from persons that had screened negative for HIV and hepatitis B viruses and had no reported history of international travel in the six previous months.

For the area of low *P. falciparum* endemicity, a tracking results continuously (TRaC) survey was conducted in 2012 as part of Global Fund’s Round 8 activities that builds on the Strategic Plan against Malaria in Haiti. A 2011 nationwide survey reported Haiti to have a low national parasite prevalence of less than 1 % as estimated by PCR [16]. The 2012 survey was cross-sectional and implemented by Population Services International (PSI) utilizing a community-based household design in collaboration with Haiti’s Ministry of Health (MSPP) and the Centers for Disease Control and Prevention (CDC). Dried blood spots (DBS) were obtained for each individual consenting to participate by spotting finger-prick blood onto Whatman 903 Protein Saver Cards or Whatman 1 circular filter paper (GE Healthcare, Piscataqway, NJ, USA), and air drying for at least 2 h. Samples were individually packaged into plastic bags with desiccant and stored at 4 °C prior to analysis. For this study, 580 samples were randomly selected which represented 12 % of the TRaC 2012 sample population. In line with previous national estimates, this subset of samples was found to have a parasite prevalence of under 1 %.

### Blood spot elution and immunoassays

A 6 mm circular punch was taken from the centre of each blood spot, corresponding to 14 μL whole blood, for elution. Samples were shaken overnight at room temperature in 140 μL protein elution buffer containing: PBS (pH 7.2), 0.05 % Tween-20, 0.05 % sodium azide, and stored at 4 °C until analysis. Elution from blood spots provided an initial 1:10 dilution, and samples were further diluted 1:40 in ELISA or Luminex sample diluent for a final whole blood dilution of 1:400, corresponding to a serum dilution of approximately 1:800 with the assumption of 50 % haematocrit in whole blood. Three *P. falciparum* merozoite surface protein 1 (MSP-1) fragments were employed, including two allelic forms of the 42kD fragment of MSP-1: MSP-1p42(D) and MSP-1p42(F) from the 3D7 and FVO strains, respectively, and one form of the 19 kD fragment (MSP-1p19) fused to glutathione S-transferase (GST) cloned from *P. falciparum* isolate 3D7. The sequence encoding the MSP-1p19 fragment previously described by Burghaus and Holder [17] was PCR amplified from 3D7 parasite strain genomic DNA using the following forward and reverse deoxyoligonucleotides: 5′-CGC GGA TCC AAC ATT TCA CAA CAC CAA TGC GTA-3′ and 5′-GCG GAA TTC*TTA* GTT AGA GGA ACT GCA GAA AAT ACC-3′, respectively. The *Bam*HI and *Eco*RI restriction endonuclease sites used for directional cloning into pGEX 4T-2 (GE Healthcare) are underlined in the sequences, and an in-frame stop codon (italics) was included in the reverse primer. Standard protocols for PCR amplification, cloning into BL21 strain *Escherichia coli* (Stratagene, LaJolla, CA), expression, and purification of a GST fusion protein product have previously been reported [18, 19]. Fusion protein eluted from the glutathione Sepharose 4B affinity column (GE Healthcare) was dialyzed against 25 mM Tris buffer at pH 7.5 as previously described [20] and loaded onto a MonoQ HR 5/5 strong anion exchange column (GE Healthcare) previously equilibrated with 25 mM Tris buffer at pH 7.5. The column was washed up to 0.2 M NaCl in 25 mM Tris buffer at a flow rate of 1 mL/min, and then the bound GST-MSP-1p19 fusion protein was eluted with a 5 min linear gradient from 0.2 to 0.5 M NaCl in Tris buffer. The fractions that included a large absorbance peak centered at ~0.3 M NaCl were combined, dialyzed overnight at 4 °C against 500 volumes of buffer containing 0.85 % NaCl and 10 mM Na_2_HPO_4_ at pH 7.2 (PBS) (Spectra/Por 3 dialysis membrane, 3500 Da cutoff, Spectrum Laboratories, Rancho Dominguez, CA, USA), and concentrated using a Centricon-10 centrifugal filter device (Millipore Corporation, Bedford, MA, USA). The final concentration of the GST-MSP-1p19 fusion protein was >0.5 mg/mL (BCA microassay, Pierce, Rockford, IL, USA) with a yield of approximately 0.25 mg/L of *E. coli* culture. The external domain of Apical Membrane Antigen-1 (AMA-1) and MSP-1p42 antigens were produced at Walter Reed Army Institute of Research (WRAIR) under previously published conditions [21].

Before analysis, ELISA assays were optimized for antigen coating concentration and dilution of horseradish peroxidase (HRP) conjugate to reduce background signal while still providing strong detection of the specified IgG. After optimization, all samples were run under the same conditions using a standard in-house protocol. Briefly, Immulon 2 HB plates (ThermoFisher Scientific, Waltham, MA, USA) were coated with 100 μL of 0.25 μg/mL antigen in PBS overnight at 4 °C and then blocked for 2 h the next day with 5 % non-fat milk in PBS, 0.05 % Tween-20 (PBS-T, Sigma-Aldrich, St. Louis, MO, USA). Samples were diluted in blocking buffer, added in duplicate at 100 μL/well, and incubated for 2 h with gentle shaking. An HRP-conjugated secondary antibody (goat anti-human IgG) was diluted 1:12,000 in PBS-T, and 100 μL added for 1 h. For each well, 100 μL peroxidase substrate (KPL, West Chester, PA, USA) was added and color allowed to develop for 10 min. The reaction was stopped by adding 100 μL 0.2 M H_2_SO_4_ and plates read on a spectrophotometer (Molecular Devices SpectraMAX, Sunnyvale, CA, USA) at 450 nm. Positive and negative control wells were included on each plate to ensure consistency between readings. At the end of the study, any particular plate was re-run if its controls showed 25 % or more variation among all plates performed for the study.

The same DBS elutions were also assayed using bead-based multiplex technology. Antigens were coupled to BioPlex^®^ COOH beads (BioRad, Hercules, CA, USA) according to manufacturer’s protocol in the presence of 50 mM 2-(4-morpholino)-ethane sulfonic acid, 0.85 % NaCl at pH 5.0 and an antigen concentration of 20 μg/mL for all antigens. Sulfo-NHS was purchased from ThermoFisher and EDC from Sigma-Aldrich. As a control to test for any serum IgG against GST for the GST-MSP-1p19 fusion protein, a bead was included in the panel which was coupled to GST. Samples were diluted in blocking buffer containing 0.5 % Polyvinyl alcohol (Sigma), 0.8 % Polyvinylpyrrolidine (Sigma), 0.1 % casein (ThermoFisher), 0.5 % BSA (Millipore), 0.3 % Tween-20, 0.1 % sodium azide, and 0.01 % *E. coli* extract to prevent non-specific binding. Reagent diluent (Buffer C) consisted of PBS-T plus 0.5 % BSA, 0.02 % sodium azide. Filter bottom plates (Multiscreen 1.2 μm, Millipore) were pre-wetted with PBS-T and 1500 beads/analyte incubated with sample in duplicate for 1.5 h under gentle shaking. Secondary antibodies tagged with biotin (1:500 anti-human IgG_1–3_, Southern Biotech, Birmingham, AL, USA; 1:2500 anti-human IgG_4_, Sigma) were incubated for 45 min, and subsequent incubation with streptavidin–phycoerythrin (1:200, Invitrogen) for 30 min. Plates had a final wash incubation with reagent diluent for 30 min and were read on a Bio-Plex 200 machine by generating the median fluorescence signal for 50 beads/analyte and then the mean fluorescence intensity (MFI) between the duplicate wells. Final MFI was reported for a sample after subtracting MFI values from blank background beads that were included on each plate.

### Statistical analyses

Data were fitted to parametric distributions by statistical software programs SAS 9.3, StataSE 13, and R 3.1.2 and means and standard deviations estimated. Statistical appropriateness of fitted distributions was performed in the SAS software package with the PROC UNIVARIATE procedure and HISTOGRAM statement. The null hypothesis of the Anderson–Darling, Cramér–von Mises, and Kolmogorov–Smirnov tests is that the data of interest are appropriate for the distribution of interest, so a *P* value of >0.05 would indicate a statistically-acceptable fit. The analysis relaxed the stringency to a *P* value of >0.01 in order to be inclusive for more tests for the purpose of comparisons between immunoassays and among antigens. When considering malaria-naive US residents as a reference population, extreme outliers could significantly affect the statistical nature of distributions, so any optical density value (OD) >0.3 or MFI >250 was eliminated from the ‘true negative’ analysis. The number of removals for each antigen and each immunoassay is listed in Additional file 1. No individual had more than one value removed for readings on all antigens.

Finite mixture regression models were fitted to the dataset by StataSE’s *fmm* and R’s *flexmix* command, as well as SAS’s PROC FMM. All use maximum likelihood estimation to fit an unweighted two-component model of ‘seronegatives’ and ‘seropositives’. The mean and standard deviation were collected from the seronegative component and used for further statistical analysis. Seroprevalence curves and estimates for seroconversion and seroreversion rates were based on fitting data to a reversible catalytic model as described previously [6].

Continuous data were fitted to penalized B-splines for both ELISA and Luminex assays. A B-spline allows a defined number of connected polynomial functions to be fitted to a continuous variable. A cubic function (degree of 3) with 50 knots was used to generate each spline and a very high penalty function (λ = 1,000,000) was imposed to prevent high variation between knots and allow curve smoothing.

## Results

Since readings for immunoassays can vary widely depending on reagents and protocols, it was investigated how ELISA and multiplex assays performed when the same dilution series was ran using the specified reagents and protocols that would be employed for the entire study. As MSP-1 and AMA-1 antigens are thought to induce robust and long-lived humoral immune responses after multiple exposures to malaria infection [8, 22, 23], blood was pooled from individuals residing in a Kenya region holoendemic for *P. falciparum* malaria, spotted onto filter paper, eluted, and titrated using each immunoassay. Through the ELISA assay, high IgG concentrations in Kenyan blood allowed samples to be diluted to 1:10^5^-fold before reaching the lower asymptote of the sigmoidal curve (Additional file 2), but the multiplex assay allowed further titration capacity. This observation has been reported in other falciparum malaria studies, which have found bead-based multiplex assays provided higher titration capacity when directly compared with ELISA [24, 25]. Possible explanations for this finding include the 3-dimensional nature of the bead assay as well as the ability of flow cytometry emission detectors to read a wide range of fluorescence intensities.

**Fig. 1:**
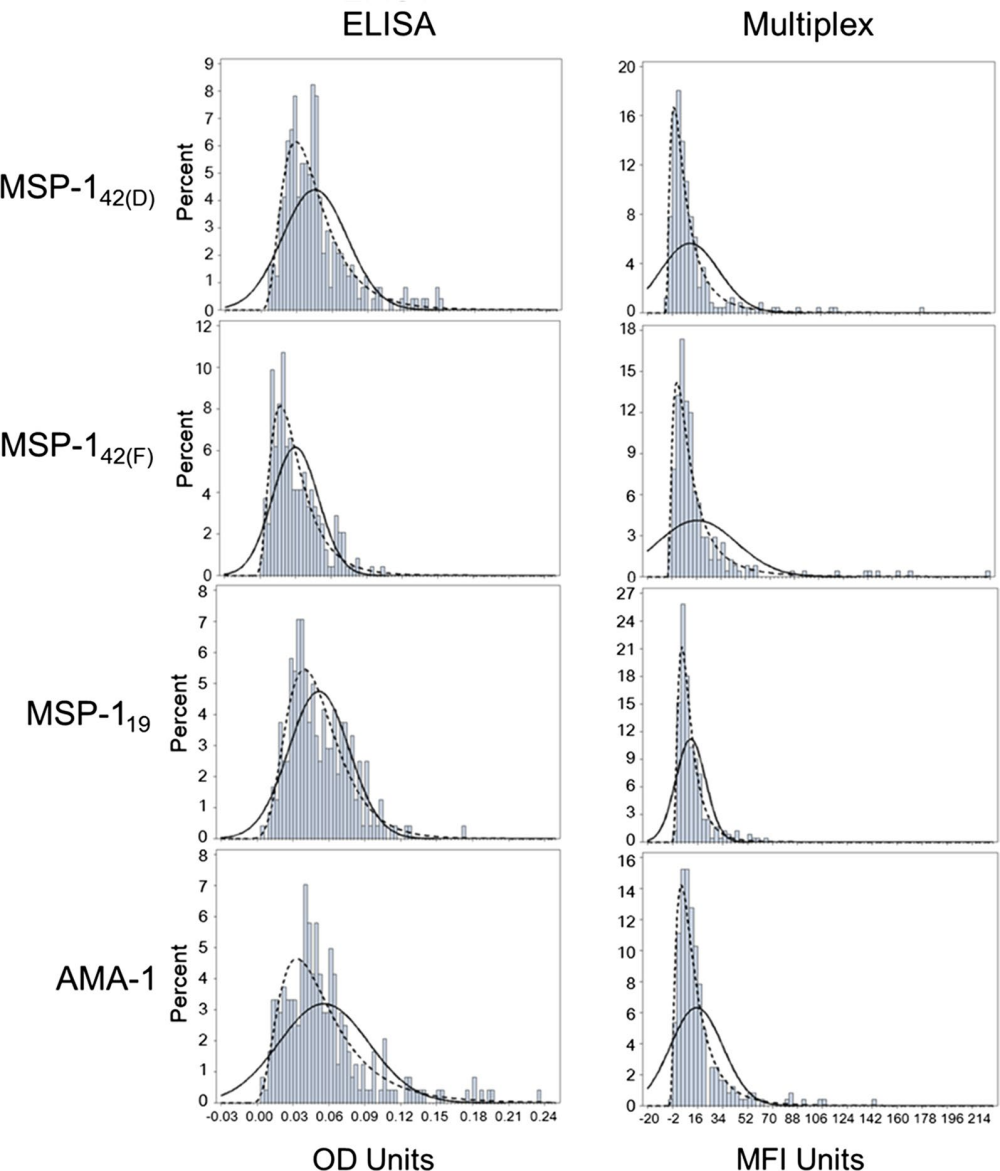
Histograms of samples from malaria-naive persons (n = 247) as analysed by ELISA and Luminex assays. Charts are overlaid with normal (*solid line*) and lognormal (*dashed line*) fitted curves. The x-axes are truncated to the upper limit of the observed data: OD units, 0.25; MFI units, 240

One of the common approaches to analysing serological data includes classifying persons in a sample as either ‘seropositive’ or ‘seronegative’ depending upon IgG titres to a specific *P. falciparum* antigen. This useful method can simplify interpretation and allow direct comparisons among sampling areas [6, 9] or time points [26] of interest. However, due to the inherent background signal from a blood or serum sample, multiple methods have been devised in order to determine at which signal intensity an individual should be considered seropositive [6, 14, 27, 28]. A reference population is commonly used to determine the whole blood, serum, or DBS elution background signal for the immunoassay, reagents, and protocol in practice. Ideally, this reference population would be derived at the sampling site from individuals known to never have been exposed to malaria, but since this is practically impossible, Europeans or Americans with no history of travel to malaria endemic countries typically act as the non-immune reference. For this study, 247 US residents were used as a non-immune reference to indicate the ‘true negative’ sero-distribution for each antigen. Distributions for both immunoassays and all four antigens showed a very similar pattern of positively-skewed data which visually fit poorly to normal (Gaussian) distribution (Fig. 1). When attempting to fit the data to various parametric distributions which assume a positive skew, it was found that ELISA data were statistically appropriate for many distributions, whereas Multiplex data were not (Additional file 3). This is likely due to the high positive values for both kurtosis and skewness for the Multiplex data. Notably, the normal distribution was not an appropriate statistical fit for any of the malaria-naive data on either immunoassay. As the lognormal distribution was found to be most appropriate for ELISA data, and showed a good visual fit to Multiplex data (Fig. 1), this was the only non-normal distribution of interest for the remainder of the study.

**Fig. 2:**
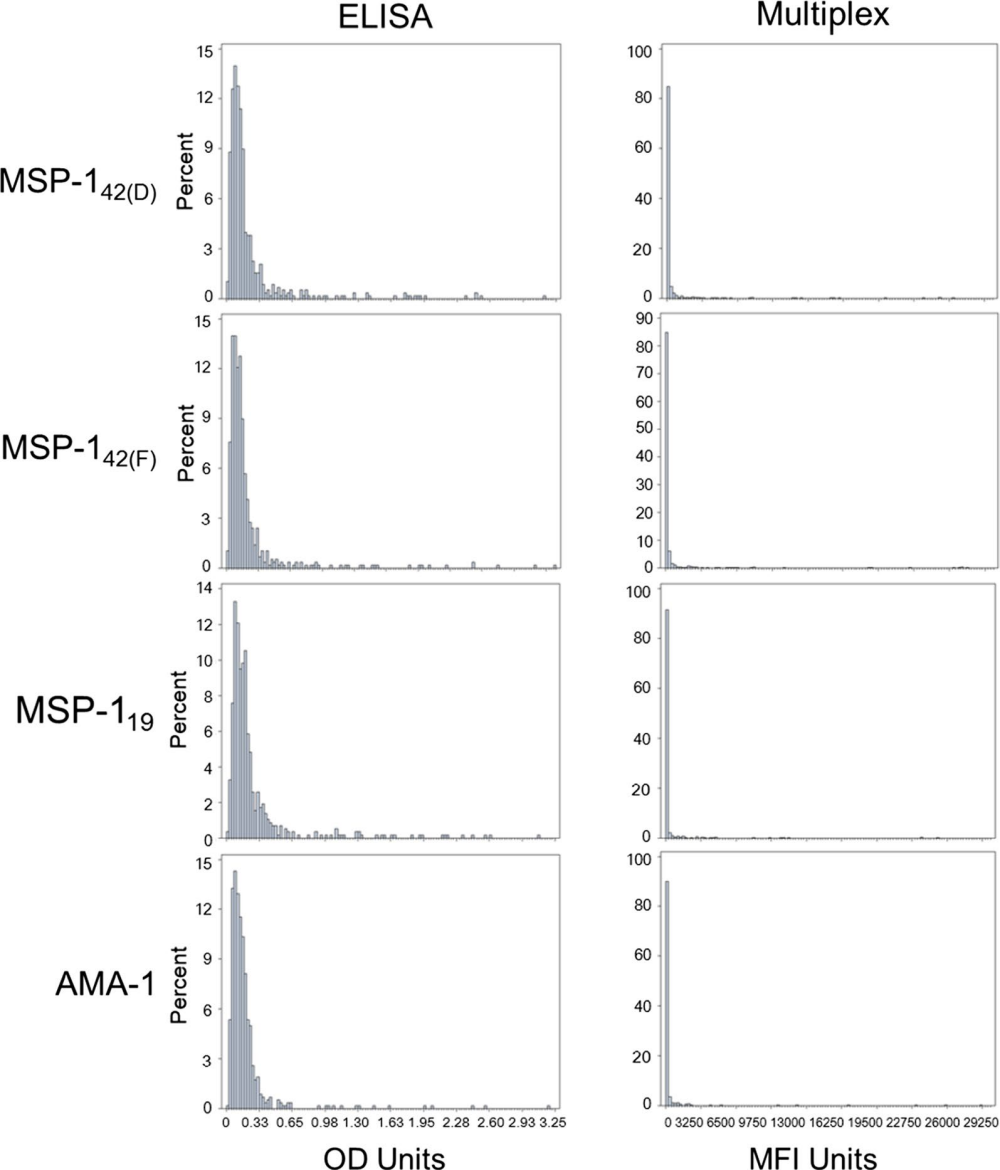
Histograms of samples (n = 580) from a region of low-endemic *P. falciparum* transmission. The x-axes display the entire dynamic range of each assay. OD units, 0–3.5; MFI units, 0–30,000

**Fig. 3:**
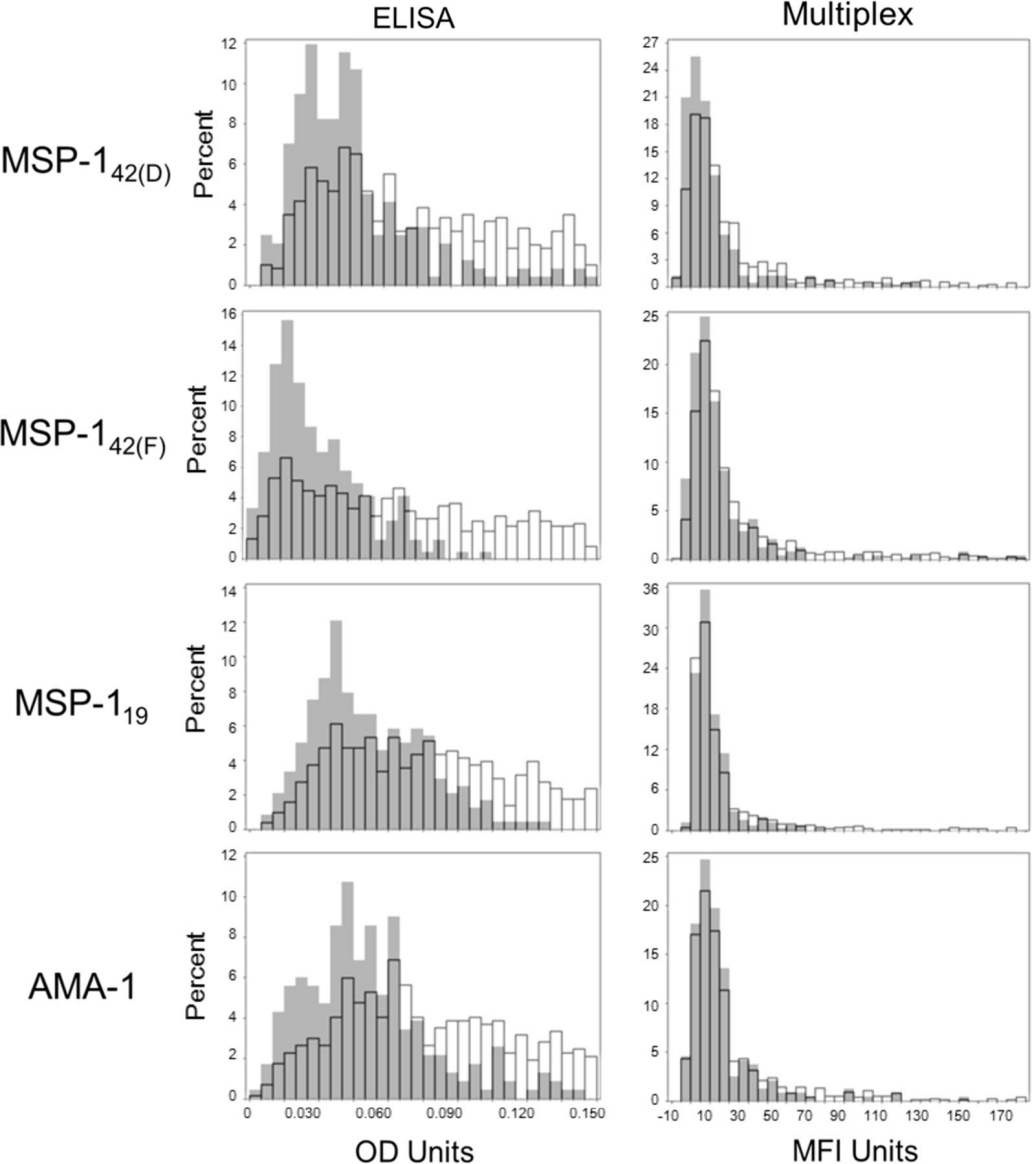
Overlay of histogram from malaria-naive population (*grey bars*), on sample population (*open bars*). The x-axis for both immunoassays is truncated as in Fig. 1 to show comparative overlay

Inspection of the immunoassay signal distribution for persons from an area of low *P. falciparum* transmission showed a very similar depiction with a large percentage of the sample population congregating on the low end of the x-axis (Fig. 2), a finding also seen in other studies [27, 29, 30]. Direct comparison of an individual’s signal for both immunoassays yielded a strong positive correlation and high agreement between assays (Additional file 4). The initial method of determining a seropositivity cut-off utilized the ‘classical approach’ [28] with malaria-naive population as a reference group and the mean plus standard deviations providing a cutoff value. Overlay of histograms from Fig. 1 (malaria-naive) onto Fig. 2 (sample population) showed clear overlap in the highest density of the sample distributions for the Multiplex assay with less than five MFI units separating the peaks of the two histograms (Fig. 3). However, the overlay of ELISA histograms did not show a similar pattern, indicating that malaria-naive US residents were likely a less suitable reference population for ELISA data. A key assumption in using the serodistribution of a malaria-naive population is that their blood/serum is representative of the sample population—just lacking IgG antibodies against malaria antigens. If using a malaria-naive population as a reference, an adequate panel of samples should be run to visually inspect the overlap between the sample and reference populations. Data presented in this study specifically provides comparison between populations in a malaria-naive and low-endemic settings using specific assay conditions for ELISA and Multiplex (see “Methods”). Previously published studies utilizing different assay variations to determine seropositivity cutoffs for sample populations (of varying endemicity) may have found US or European residents to be an appropriate malaria-naive population for that particular study. To this point, it is of value to statistically investigate a malaria-naive reference population under each study’s particular conditions. For this study’s malaria-naive population, the mean signal and standard deviations for all four antigens were generated for both immunoassays and for both normal and lognormal distributions (Table 1). Frequently, the mean signal plus three or five standard deviations is designated as a seropositivity cutoff with a high degree of confidence [6, 14, 31]. When comparing normal to lognormal distributions, ELISA data gave almost identical cutoff values, where Multiplex cutoff values were slightly more conservative under a normal distribution.

**Table 1:** Means, standard deviations, and seropositivity cutoff values for each antigen as determined by different immunoassays and statistical methods

Distribution, assay	Antigen	Malaria-naïve reference population			Finite mixture model, first component		
		Mean (SD)	Mean + 3SD	Mean + 5SD	Mean (SD)	Mean + 3SD	Mean + 5SD
Normal distribution							
ELISA (in OD)	MSP1-42(D)	0.045 (0.027)	0.126	0.180	0.125 (0.069)	0.332	0.470
	MSP1-42(F)	0.030 (0.019)	0.087	0.125	0.127 (0.068)	0.331	0.467
	MSP1-19	0.052 (0.025)	0.127	0.177	0.157 (0.080)	0.397	0.557
	AMA-1	0.056 (0.038)	0.170	0.246	0.14 (0.069)	0.347	0.485
Multiplex (in MFI)	MSP1-42(D)	10.7 (21.2)	74.3	116.7	18.3 (16.7)	68.4	101.8
	MSP1-42(F)	16.4 (29.1)	103.7	161.9	21.7 (22.4)	88.9	133.7
	MSP1-19	11.1 (10.7)	43.2	64.6	11.4 (10.5)	42.9	63.9
	AMA-1	15.3 (19.0)	72.3	110.3	21.7 (23.5)	92.2	139.2
Lognormal distribution							
ELISA (in OD)	MSP1-42(D)	0.045 (0.027)	0.126	0.180	0.085 (0.572)	1.801	2.945
	MSP1-42(F)	0.030 (0.021)	0.093	0.135	−1.214 (1.242)	2.512	4.996
	MSP1-19	0.052 (0.026)	0.130	0.182	0.377 (0.385)	1.532	2.302
	AMA-1	0.056 (0.037)	0.167	0.241	0.292 (0.500)	1.792	2.792
Multiplex (in MFI)	MSP1-42(D)	10.1 (17.0)	61.1	95.1	2.5 (0.96)	5.4	7.3
	MSP1-42(F)	15.3 (21.6)	80.1	123.3	2.5 (0.69)	4.6	6.0
	MSP1-19	10.9 (9.8)	40.3	59.9	2.0 (0.84)	4.5	6.2
	AMA-1	15.0 (16.6)	64.8	98.0	2.5 (0.91)	5.2	7.1

An additional strategy for determining a cutoff value is the implementation of the finite mixture regression model (FMM), which aims to identify an indicated mixture of densities using maximum likelihood estimation (MLE). Normal and lognormal distributions were specified for each antigen’s data and attempted to fit to a two-component mixture (Additional file 5). The FMM ELISA cutoff values were much higher under both distributions when compared with values garnered from the malaria-naive population (Table 1). Interestingly, FMM cutoff values were only slightly less conservative for Multiplex data under a normal distribution when compared to the malaria-naive population, but quite lower under a lognormal distribution. An innate assumption of finite mixture model regression is that the number of true components is known a priori [32], even if a parametric distribution is not assumed. In biological reality, it is unlikely that only two components exist: a seronegative and a seropositive, if true definable components exist at all. Numerous true distributions may exist depending on genotype (host and parasite), age of infection, and co-infections, among many other factors. As the seropositivity cutoff values varied greatly depending on statistical method used, the amount and percentage of the sample population that would be considered ‘seropositive’ also varied (Table 2). Since all three MSP-1 fragments showed high correlation among each other in signal intensity, the MSP-1p19 antigen estimates are used as the MSP-1 representative.

**Table 2:** Number and percentage of sample population (n = 580) considered seropositive for MSP-1-19 and AMA-1 antigens when applying different cutoff criterion

Assay	Antigen	Malaria-naïve reference population		Finite mixture model	
		Mean + 3SD	Mean + 5SD	Mean + 3SD	Mean + 5SD
Normal distribution					
ELISA	MSP-1	362 (62.4 %)	251 (43.3 %)	66 (11.4 %)	41 (7.1 %)
	AMA-1	197 (34.0 %)	81 (14.0 %)	38 (6.6 %)	25 (4.3 %)
Multiplex	MSP-1	126 (21.7 %)	105 (18.1 %)	126 (21.7 %)	105 (18.1 %)
	AMA-1	108 (18.6 %)	76 (13.1 %)	90 (15.5 %)	71 (12.2 %)
Lognormal distribution					
ELISA	MSP-1	355 (61.2 %)	238 (41.0 %)	15 (2.6 %)	5 (0.9 %)
	AMA-1	200 (34.5 %)	87 (15.0 %)	5 (0.9 %)	1 (0.2 %)
Multiplex	MSP-1	130 (22.4 %)	110 (19.0 %)	451 (77.8 %)	403 (69.5 %)
	AMA-1	120 (20.7 %)	85 (14.7 %)	468 (80.7 %)	421 (72.6 %)

**Fig. 4:**
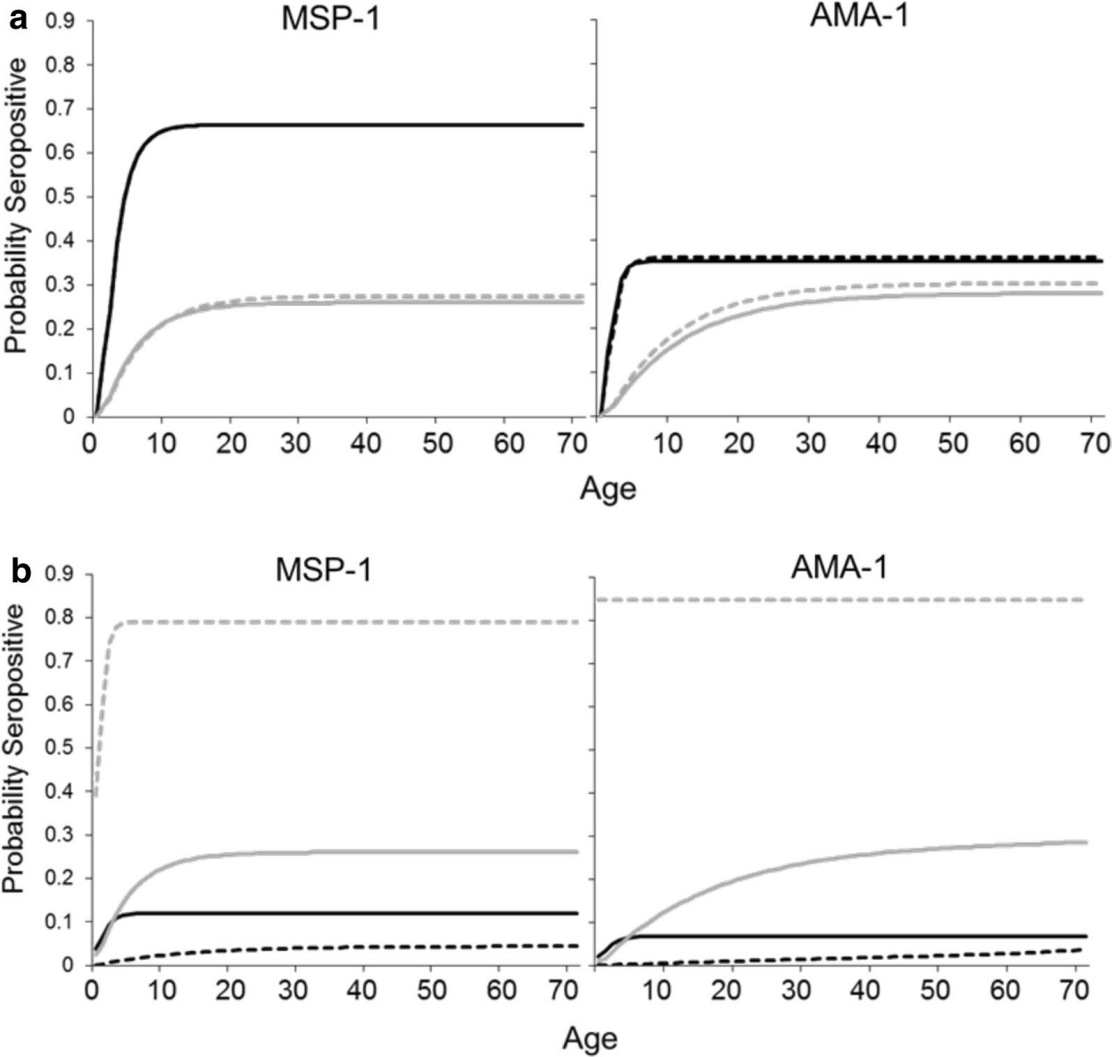
Seroprevalence curves for MSP-1 and AMA-1 antigens based on different immunoassays, fitted distributions, and seropositive cutoff determinations. The MSP-1p19 antigen was chosen to represent MSP-1 response as a whole. Curves represent ELISA (*black lines*) and multiplex assays (*grey lines*) with normal (*solid lines*) and lognormal (*dashed lines*) distributions. Seropositivity was based on mean + 3SD cutoff value for all comparisons. **a** Curves generated when using malaria-naive persons as reference. *Note* ELISA normal and lognormal seroprevalence curves for the MSP-1 antigen overlap. **b** Curves generated by finite mixture model approaches

When seropositivity is coupled with age data for a sample population, estimates are able to be calculated for the rates of acquiring (seroconversion) or loss of (seroreversion) IgG antibodies against a pathogen by fitting data to a reversible catalytic conversion model [6, 9, 33]. Younger age groups are important for this model, as the first years of life represent the most dynamic period of antibody acquisition. For falciparum malaria, seroconversion rates have been shown to correlate with entomological inoculation rates [6], and diminish with successful malaria control interventions [5, 34]. When using the malaria-naive population as a reference, seroprevalence curves generated by the catalytic model tended to vary more by immunoassay, rather than which parametric distribution was specified (Fig. 4a). This finding was also reflected in the estimated seroconversion (λ) and seroreversion (ρ) rates (Additional file 6). When moving to a higher stringency of mean plus five standard deviation seropositivity cutoff, ELISA estimates were greatly reduced, whereas Multiplex estimates largely remained unchanged. When the FMM strategy was used to generate seropositivity cutoff values, different parametric distributions gave dissimilar results for seroprevalence curves (Fig. 4b) and sero-rates (Additional file 6). Although the FMM approach has shown to be quite useful in areas of varying degrees of *P. falciparum* transmission, potential disadvantages of this method in settings of very low historical transmission include the high overlap between seropositive and seronegative populations (illustrated in [28]) and estimates of seroreversion rates that are higher than the estimated seroconversion rate. Methods for determining seropositivity cutoffs become more of a moot point when comparisons are made only within a defined study where the same immunoassay, reagents, and protocols are employed for all samples. In fact, it may be suitable to choose any value within the ‘indeterminate range’ [33] for comparisons within a single study, as long as no evidence exists that different people groups have dissimilar background signal intensities. When applying new technology or techniques to an area which has already had multiple sero-surveys, it may be necessary to not apply too much weight to the magnitude of the ‘percent seropositive’ or seroconversion rate estimates, but rather provide relative comparisons among different regions within a geographical area. To truly compare estimates among different malaria sero-studies, an international serological gold standard may be appropriate, as is the practice for various other diseases [35–37]. A gold standard serum could also provide a means by which to calibrate a research group’s unique immunoassay to designate the seropositivity cutoff of the OD or MFI value at ‘X’ international units.

**Fig. 5:**
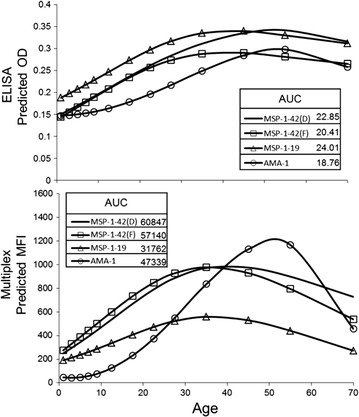
Penalized B-splines fitted to continuous data for each antigen MSP-1p42(D) *no markers*; MSP-1p42(F) *square markers*; MSP-1p19 *triangle markers*; AMA-1 *circle markers*. *Inset box* displays area under the curve (AUC) calculations for each antigen by each assay

Since transforming continuous data to binary results in an inherent loss of information, data were also analysed by maintaining immunoassay signal intensities. High IgG titres against *P. falciparum* antigens would be the likely result of active or recent parasite exposure, or evidence of multiple exposures throughout life [38]. Penalized B-splines were fitted for each antigen within each immunoassay, and area under the curve (AUC) calculated (Fig. 5). The shapes of all B-splines were very similar, with increases in the predicted signal intensity as persons progress through the first 30 years of life, followed slight declines in the 50s and 60s. A decrease in IgG titres for these ages has been observed elsewhere for the AMA-1 antigen [39], but MSP-1 serology has shown maintenance of high titres throughout life. By percentage, AUC values within the ELISA assay were similar, with the AMA-1 curve showing the greatest AUC deviation from the mean of 13 %. Multiplex B-splines were much more varied with the MSP-1p19 spline showing the greatest AUC deviation of 36 % from the mean. Due to the integral differences between the two immunoassays, AUC comparisons would be incompatible, but assessments within an assay of different time points or geographical areas could potentially yield productive comparisons.

## Conclusions

Malaria serological data have shown great potential for the elucidation of varying transmission regions within an area. Numerous *Plasmodium* and vector antigens have been well characterized, and multivariate approaches to malaria serology may yield robust analyses in the future. As *P. falciparum* exposure incidence is reduced in a population, levels of IgG against falciparum will reduce as well, and defining what constitutes an individual as seropositive becomes more difficult. Here, direct comparisons are presented of multiple methods for acquiring and analysing falciparum serological data from an area of low *P. falciparum* endemicity. It was found that the Multiplex bead assay provided a higher titration capacity, and a denser histogram of data when used to generate assay from a low-endemic sample population. Additional analyses found that a malaria-naive sample set was an appropriate reference when using a bead-based assay and that ELISA estimates for seropositives within a population varied greatly depending on the method used to develop a seropositivity cutoff value. The novel method of applying B-splines to serological data provides an additional technique for displaying this type information.

## Additional files

10.1186/s12936-015-0955-1Table: Extreme outliers removed from malaria-naïve analysis.
10.1186/s12936-015-0955-1 Figure: Titrations *P. falciparum* hyperimmune serum as read for MSP-1p19 and AMA-1 antigens on ELISA and Luminex assays shown by tabular format (A) and titration curves (B).
10.1186/s12936-015-0955-1 Table: Distributions that were appropriate (p >0.01) by various statistical tests for ELISA and Multiplex data of malaria-naïve persons. Letter indicates appropriate fit by that test.
10.1186/s12936-015-0955-1 Figure: Scatterplots of ELISA OD (x-axis) and Multiplex MFI (y-axis) for each antigen used in the study. All axes are at log_2_ scale.
10.1186/s12936-015-0955-1 Figure: Two-component finite mixture model fitting to each antigen’s signal intensity from both immunoassays. For each immunoassay, top histograms are log_e_ transformed data and bottom histograms are non-transformed data. For seropositivity cutoffs in Table 1, the mean and standard deviation of the first (leftmost) component was used. Maximum likelihood estimation methods were unable to fit a second component to the ELISA AMA-1 data.
10.1186/s12936-015-0955-1 Table: Estimates of seroconversion (λ) and seroreversion (ρ) rates by reversible catalytic model.

## Abbreviations

AUC: area under the curve
AMA-1: apical membrane antigen 1
DBS: dried blood spot
ELISA: enzyme-linked immunosorbant assay
FMM: finite mixture model
IgG: immunoglobulin G
MLE: maximum likelihood estimation
MSP-1: merozite surface protein 1
OD: optical density
