# Supplementary material for: Multiple comparisons analysis of serological data from an area of low Plasmodium falciparum transmission
Source: Malar J. 2015 Nov 4;14:436. doi: 10.1186/s12936-015-0955-1 (PMC4634594; doi:10.1186/s12936-015-0955-1)
Supplement: Supplementary file 1 — 10.1186/s12936-015-0955-1Table: Extreme outliers removed from malaria-naïve analysis. [file 12936_2015_955_MOESM1_ESM.docx]

Additional file 1. **Extreme outliers removed from malaria-naïve analysis**

| **Antigen** | **# removed ELISA** | **# removed Multiplex** |
| --- | --- | --- |
| MSP-1-42(D) | 1 | 0 |
| MSP-1-42(F) | 1 | 2 |
| MSP-1-19 | 3 | 0 |
| AMA-1 | 2 | 1 |
