# Supplementary material for: Multiple comparisons analysis of serological data from an area of low Plasmodium falciparum transmission
Source: Malar J. 2015 Nov 4;14:436. doi: 10.1186/s12936-015-0955-1 (PMC4634594; doi:10.1186/s12936-015-0955-1)
Supplement: Supplementary file 2 — 10.1186/s12936-015-0955-1 Figure: Titrations P. falciparum hyperimmune serum as read for MSP-1p19 and AMA-1 antigens on ELISA and Luminex assays shown by tabular format (A) and titration curves (B). [file 12936_2015_955_MOESM2_ESM.docx]

Additional file 2**.**

Titrations *P. falciparum* hyperimmune serum as read for MSP-1p19 and AMA-1 antigens on ELISA and Luminex assays shown by tabular format (A) and titration curves (B)

**
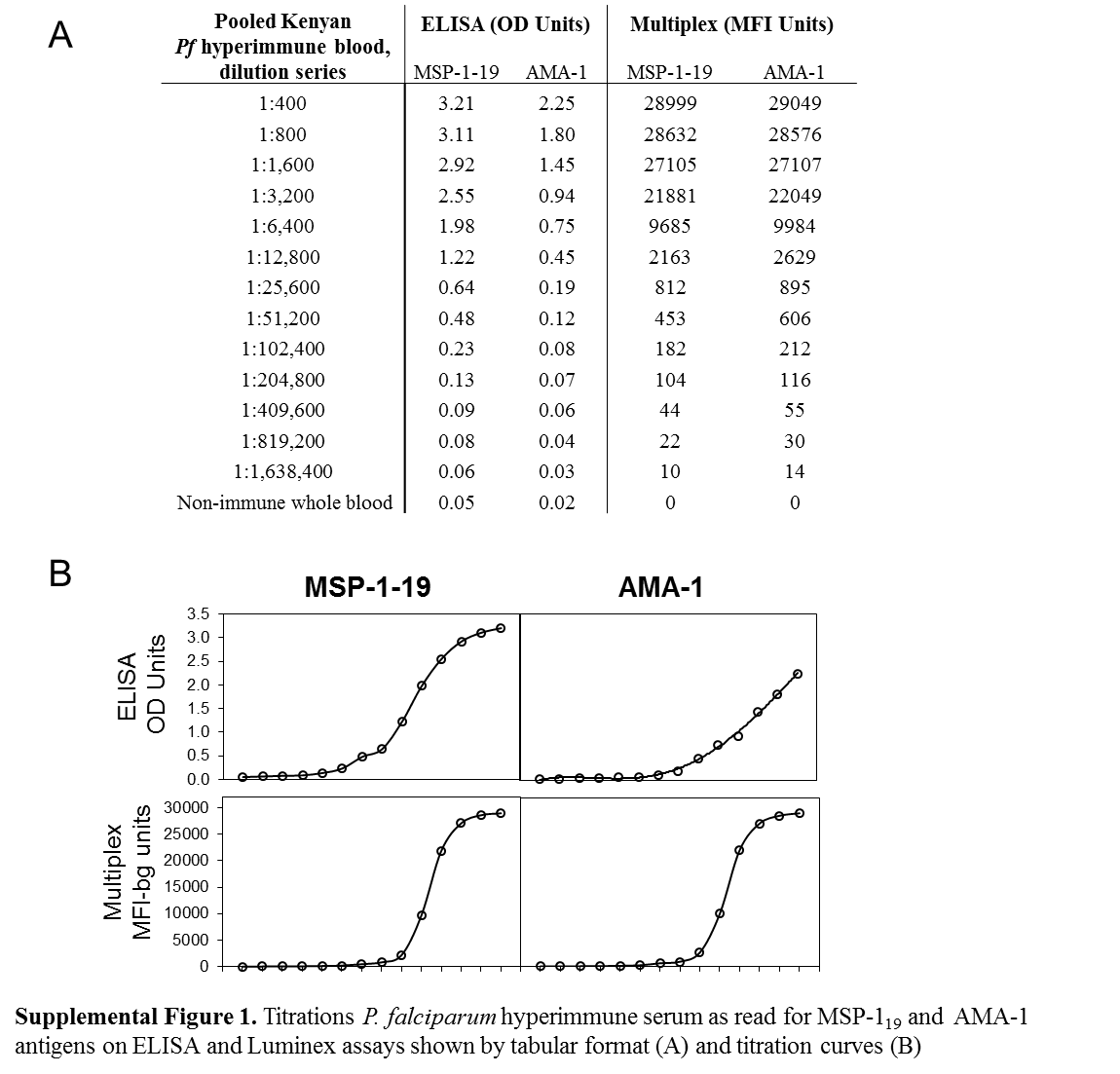
**
