# Supplementary material for: Multiple comparisons analysis of serological data from an area of low Plasmodium falciparum transmission
Source: Malar J. 2015 Nov 4;14:436. doi: 10.1186/s12936-015-0955-1 (PMC4634594; doi:10.1186/s12936-015-0955-1)
Supplement: Supplementary file 3 — 10.1186/s12936-015-0955-1 Table: Distributions that were appropriate (p >0.01) by various statistical tests for ELISA and Multiplex data of malaria-naïve persons. Letter indicates appropriate fit by that test. [file 12936_2015_955_MOESM3_ESM.docx]

Additional file 3**. Distributions that were appropriate (p >0.01) by various statistical tests for ELISA and Multiplex data of malaria-naïve persons. Letter indicates appropriate fit by that test.**

|  |  | **ELISA** |  |  |  | **Multiplex** |  |  |
| --- | --- | --- | --- | --- | --- | --- | --- | --- |
| **Distribution** | **MSP1-42(D)** | **MSP1-42(F)** | **MSP1-19** | **AMA-1** | **MSP1-42(D)** | **MSP1-42(F)** | **MSP1-19** | **AMA-1** |
| Normal |  |  |  |  |  |  |  |  |
| Lognormal | A,C,K | A,C,K | A,C,K | A,C,K |  |  |  |  |
| Weibull* |  | A,C | A,C |  |  |  |  |  |
| Gamma |  | A,C,K | A,C,K |  |  |  |  |  |
| Rayleigh |  |  | A,C,K |  |  |  |  |  |
| Gumbel | C |  | A,C,K | C,K |  |  |  |  |
| Inv. Gaussian | C,K |  | K |  |  |  | C |  |

*, only Anderson-Darling and Cramer-von Mises statistical tests are available for Weibull distribution

A: Anderson-Darling test

C: Cramer-von Mises test

K: Kolmogorov-Smirnov test
