# Supplementary material for: Multiple comparisons analysis of serological data from an area of low Plasmodium falciparum transmission
Source: Malar J. 2015 Nov 4;14:436. doi: 10.1186/s12936-015-0955-1 (PMC4634594; doi:10.1186/s12936-015-0955-1)
Supplement: Supplementary file 4 — 10.1186/s12936-015-0955-1 Figure: Scatterplots of ELISA OD (x-axis) and Multiplex MFI (y-axis) for each antigen used in the study. All axes are at log2 scale. [file 12936_2015_955_MOESM4_ESM.docx]

Additional file 4**.**

Scatterplots of ELISA OD (x-axis) and Multiplex MFI (y-axis) for each antigen used in the study. All axes are at log_2_ scale.

**
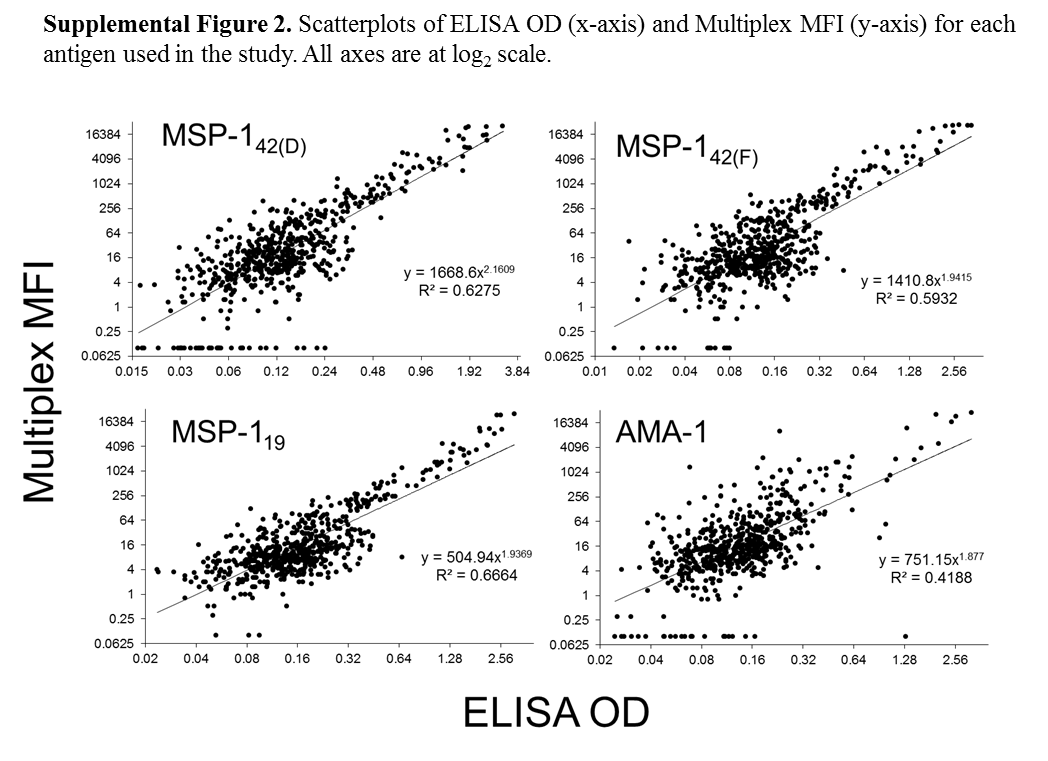
**
