# Supplementary material for: Multiple comparisons analysis of serological data from an area of low Plasmodium falciparum transmission
Source: Malar J. 2015 Nov 4;14:436. doi: 10.1186/s12936-015-0955-1 (PMC4634594; doi:10.1186/s12936-015-0955-1)
Supplement: Supplementary file 5 — 10.1186/s12936-015-0955-1 Figure: Two-component finite mixture model fitting to each antigen’s signal intensity from both immunoassays. For each immunoassay, top histograms are loge transformed data and bottom histograms are non-transformed data. For seropositivity cutoffs in Table 1, the mean and standard deviation of the first (leftmost) component was used. Maximum likelihood estimation methods were unable to fit a second component to the ELISA AMA-1 data. [file 12936_2015_955_MOESM5_ESM.docx]

Additional file 5**.**

Two-component finite mixture model fitting to each antigen’s signal intensity from both immunoassays. For each immunoassay, top histograms are log_e_ transformed data and bottom histograms are non-transformed data. For seropositivity cutoffs in Table 1, the mean and standard deviation of the first (leftmost) component was used. Maximum likelihood estimation methods were unable to fit a second component to the ELISA AMA-1 data.

**
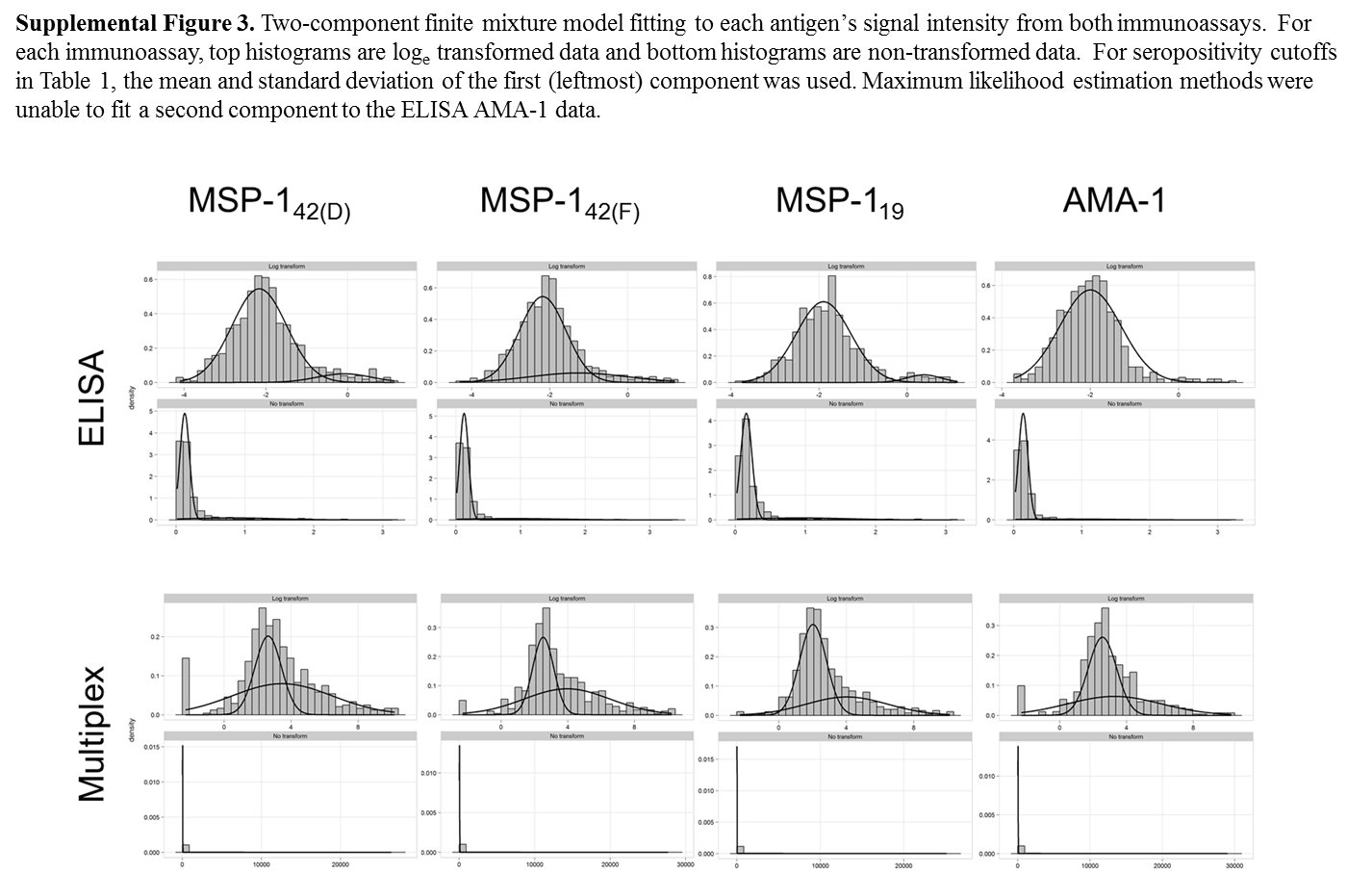
**
