# Supplementary material for: Multiple comparisons analysis of serological data from an area of low Plasmodium falciparum transmission
Source: Malar J. 2015 Nov 4;14:436. doi: 10.1186/s12936-015-0955-1 (PMC4634594; doi:10.1186/s12936-015-0955-1)
Supplement: Supplementary file 6 — 10.1186/s12936-015-0955-1 Table: Estimates of seroconversion (λ) and seroreversion (ρ) rates by reversible catalytic model. [file 12936_2015_955_MOESM6_ESM.docx]

Additional file 6**. Estimates of seroconversion (λ) and seroreversion (ρ) rates by reversible catalytic model.**

|  |  | **Malaria-naïve reference population** | | **Finite Mixture Model** | |
| --- | --- | --- | --- | --- | --- |
| **Assay** | **Antigen** | **Mean+3sd** | **Mean+5sd** | **Mean+3sd** | **Mean+5sd** |
| **Normal Distribution** | | λ (ρ) | λ (ρ) | λ (ρ) | λ (ρ) |
| **ELISA** | MSP-1 | 0.305 (0.145) | 0.152 (0.163) | 0.090 (0.659) | 0.015 (0.142) |
|  | AMA-1 | 0.380 (0.700) | 0.275 (1.65) | 0.045 (0.627) | 0.004 (0.056) |
| **Multiplex** | MSP-1 | 0.051 (0.144) | 0.047 (0.175) | 0.051 (0.146) | 0.048 (0.178) |
|  | AMA-1 | 0.026 (0.066) | 0.012 (0.032) | 0.017 (0.042) | 0.011 (0.026) |
| **Lognormal Distribution** | |  |  |  |  |
| **ELISA** | MSP-1 | 0.298 (0.151) | 0.119 (0.133) | 0.003 (0.073) | 0.006 (0.656) |
|  | AMA-1 | 0.314 (0.557) | 0.156 (0.851) | 0.001 (0.004) | 0.0 (0.0) |
| **Multiplex** | MSP-1 | 0.046 (0.122) | 0.046 (0.157) | 1.079 (0.286) | 0.398 (0.133) |
|  | AMA-1 | 0.031 (0.071) | 0.025 (0.103) | 12.20 (2.171) | 0.956 (0.284) |
